# Substrate stoichiometry and microbial metabolic preference drive the divergent accumulation of plant and microbial necromass carbon in cropland soils: evidence from a short-term experiment

**DOI:** 10.3389/fmicb.2025.1619932

**Published:** 2025-07-29

**Authors:** Hongliang Wu, Luming Wang, Xiuping Liu, Wenyan Wang, Changai Lu, Wenxu Dong

**Affiliations:** ^1^Hebei Key Laboratory of Soil Ecology, Center for Agricultural Resources Research, Institute of Genetics and Developmental Biology, Chinese Academy of Sciences, Shijiazhuang, China; ^2^Institute of Agricultural Resources and Regional Planning, Chinese Academy of Agricultural Sciences, Beijing, China

**Keywords:** agricultural residues, microbial stoichiometry metabolism, lignin phenols, microbial necromass carbon, carbon and nutrient metabolism

## Abstract

Substrate input and subsequent *ex vivo* modification and *in vivo* turnover mediated by microbial systems determine the formation of soil organic carbon (C). However, the effects and mechanisms by which substrate stoichiometry (SS) adapts to microbial metabolic preferences and influences the dynamics of plant and microbial necromass C (PNC, MNC) remain unclear. Therefore, the variations and controlling factors of PNC and MNC in top- (0–20 cm) and subsoil (20–40 cm) across different SS conditions were investigated during the whole maize season. Mainly, the SS of exogenous C, nitrogen (N), phosphorus (P), and sulfur (S) meet the metabolic requirements of fungi (NPS1) and bacteria (NPS3). Results showed that compared to NPS1, NPS3 increased MNC by 5.3% in topsoil and 13.9% in subsoil, while reducing PNC by 7.0% and 16.3%, respectively. These suggested that SS matching bacterial needs could accelerate the digestion of PNC and enhanced the accumulation of MNC, especially in subsoil. Under NPS1, dominant microbial taxa (e.g., *Planctomycetota*), phosphatase, and nutrient availability were key determinants of necromass C (NC) changes, with MNC and PNC being predominantly influenced by available N and P, respectively. *Firmicutes* was particularly influential in the subsoil. Under NPS3, a more diverse bacteria, including *Proteobacteria*, was mobilized, with β-glucosidase and available N being central to NC variations. Changes in PNC were also primarily regulated by fungi, specifically *Mortierellomycota*. Our findings of the short-term experiment suggest that SS influences the digestion or accumulation of PNC and MNC by regulating nutrient availability, C and P cycling enzymes, and functional flora, and meanwhile, emphasize that SS matching bacterial requirements enhances MNC accumulation.

## 1 Introduction

Soil organic carbon (SOC) sequestration is essential for both agricultural productivity and global carbon (C) neutrality (Schmidt et al., 2011; Wang et al., 2020). Most research has highlighted that microbial components contribute over more than half of SOC in croplands, surpassing the traditionally recognized recalcitrant plant components (Wang et al., 2021). The formation and accumulation of both plant and microbial components depend on microbial metabolic pathways, substrate stoichiometry (SS), and their alignment with microbial demands (Liang et al., 2017; Zhu et al., 2022). The interplay between SS and microbial requirements drives shifts in microbial catabolism and anabolism, influencing the transformation or accumulation of plant and microbial components, and thereby impacting SOC sequestration (Kirkby et al., 2014; Liang et al., 2017). Therefore, distinguishing and quantifying plant- and microbial-derived C components is fundamental to understanding soil C sinks (Chen et al., 2021). However, few studies have systematically examined how SS preferences of different microbial groups simultaneously regulate both plant- and microbial-derived contributions to SOC accumulation.

Recent advances in research on plant necromass C (PNC) and microbial necromass C (MNC) are elucidating their roles in SOC formation and accumulation (Liang et al., 2019; Qi et al., 2023). PNC, products of microbial catabolism, partitions into both the stable C pool and labile C pool, with the latter being preferentially transferred into microbial assimilation (Chen et al., 2020; Lavallee et al., 2019). Conversely, MNC, derived from microbial assimilation, is primarily embedded into the stable C pool, especially in the form of fungal necromass C (NC) (Liang et al., 2017, 2019). Emerging evidence suggests that PNC and MNC collectively account for more than two-thirds of SOC in cropland (Chen et al., 2021; Li et al., 2024). In fertilized soil, the addition of materials with a high C-to-nitrogen (C/N) ratio lead to slow exogenous C decomposition, resulting in negligible or no accumulation of NC. In contrast, incorporating low C/N ratio materials accelerates PNC accumulation during the early stages and promotes greater MNC accumulation at later stages (Li et al., 2024; Chen et al., 2025). Concurrent assessment the variations of PNC and MNC are essential for a comprehensive understanding of SOC sequestration. However, research on the dynamics of NC in response to SS levels and microbial resource acquisition strategies remains limited.

Functional microbial taxa and associated enzymes control microbial catabolism and assimilation processes, specifically through *ex vivo* and *in vivo* pathways. Microbes employ hydrolases to directly modify the initial C source *ex vivo*, while dominant microbial taxa assimilate the C source *in vivo* (Liang et al., 2017, 2019; Zhu et al., 2020). The different resource acquisition strategies, metabolic preferences, and environmental tolerances exhibited by various microbial taxa significantly shape community composition and function, thereby influencing their adaptive adjustments (Zhu et al., 2022; Li et al., 2024). The inherent variability and complexity of microbial communities present challenges in revealing the microbial mechanisms that drive soil C processes. Grouping microorganisms into copiotrophs and oligotrophs based on their life strategies offers a useful way to link microbial activity with environmental conditions (Hu et al., 2023). This suggests that targeted regulation of microbial communities according to their growth strategies could indirectly influence SOC sequestration. Functional hydrolases are pivotal in balancing microbial biomass production with environmental resource availability, thus affecting soil C and nutrient cycling (Sinsabaugh et al., 2009; Luo et al., 2017). Enzymes such as glucosidase and phosphatase are widely involved in microbial energy and nutrient acquisition, with phosphorus acquisition enzymes being particularly significant in agroecosystems (Wu et al., 2022). However, the interplay between major microbial taxa, functional enzymes, and their roles in *ex vivo* and *in vivo* pathways is underexplored.

In agroecosystems, substrate stoichiometry (SS) and availability are highly sensitive to management practices like organic and inorganic amendments. These alterations ultimately reshape microbial communities and metabolic functions, thereby influencing SOC accumulation (Li et al., 2020; Zhu et al., 2022). Generally, the biogeochemical cycling of C, nitrogen (N), phosphorus (P), and sulfur (S) in substrate is closely linked, where lack or imbalance in any element can hinder SOC sequestration (Kirkby et al., 2014; Fang et al., 2018). Theories such as “microbial nutrient mining” and “microbial stoichiometry metabolism” provide insights into the complex interplay between microorganisms and substrates across environments varying in resource availability (Chen et al., 2014; Heuck et al., 2015). That is, the SS determines the direction and intensity of microbial catabolism and anabolism. For example, while adding C-rich straw alone accelerates SOC minerralization, combining straw with balanced fertilizers promotes SOC accumulation (Fang et al., 2018; Wu et al., 2022). Studies have since explored how regulating SS mediates the balance between C loss (priming) and sequestration (assimilation), thus influencing SOC dynamics (Kirkby et al., 2013, 2014; Zhu et al., 2022). However, microbial communities partition into copiotrophs, which dominate in nutrient-rich conditions, and oligotrophs, adapted to nutrient-poor environments. Prior studies have also pointed out that regulating SS based on the stoichiometric ratio of soil humus may not align with the actual metabolic demands of microorganisms (Kirkby et al., 2014). Hydrolases, which adapt to microbial metabolic strategies and resource availability, also play a crucial role in these interactions (Jiang et al., 2019). Recent efforts to regulate SS according to microbial biomass stoichiometry aim to satisfy the quantitative conversion of C sources (Zhu et al., 2022). This approach helps optimize management practices that meet the needs of oligotrophs or copiotrophs, revealing their impacts on NC accumulation.

Northeast China, a region contributing 25% of the national grain, has lost 0.41 Mg C ha^−1^ over the past three decades under intensive agriculture (Zhao et al., 2018; Liu et al., 2023). This study aim to investigate the pathways and regulatory factors of SOC accumulation under varying SS levels tailored to microbial preferences (stoichiometric application of C, N, P, and S) in this region. A field *in situ* experiment was conducted throughout the maize season to (a) clarify the formation dynamics of PNC and MNC and their contributions to SOC at 0–40 cm soil depth; and (b) reveal the control factors and mechanisms of NC accumulation regulated by SS. We hypothesized that: (I) SS aligned with copiotroph requirements would enhance PNC digestion and MNC entombment; and (II) P-acquiring enzymes dominant NC accumulation under SS favoring oligotrophs (SS_O_), while C-acquiring enzymes prevail under SS meeting copiotrophs (SS_C_).

## 2 Materials and methods

### 2.1 Site description

Our study was conducted from May to October 2023 at the Changtu Station of the Institute of Applied Ecology (123°57′E, 37°48′N), Chinese Academy of Sciences, China. This region is characterized by a semi-humid continental monsoon climate, with an average annual temperature of 7°C and an annual rainfall of 580 mm, predominantly occurring from July to September. The average temperature and rainfall during the growing period (May to October) are 20°C and 420 mm, respectively. The soil, classified as Phaeozem (FAO), is loamy clay with 29% sand, 40% silt, and 31% clay. The SOC, total N, P, and S concentrations in the 0–20 cm layer were 10.9, 0.90, 0.40, and 0.22 g kg^−1^, respectively. The experimental area follows an annual maize cropping system.

### 2.2 Experiment design

To optimize the conversion of exogenous C into SOC, we manipulated the SS to match the requirements ranging from oligotrophs to copiotrophs. We assumed the stoichiometric ratios of fungi (C:N:P:S = 10,000:1,034:110:94) and bacteria (C:N:P:S = 10,000:2,004:494:264) reflected substrate requirements of oligotrophs and copiotrophs based on values published in Kirkby et al. (2014) and Zhu et al. (2022). This study provided different SS levels by applying varying amounts of straw and N, P, and S (Table 1). A total of five treatment groups were established: straw-amended soil with no nutrient addition (NPS0), NPS0 with nutrient additions to meet the metabolic requirements from fungi (NPS1) to bacteria (NPS3), and a control soil (CK). The ideal humification efficiency of the added straw was set to 30%. The straw, cut into 2–5 mm pieces, was mixed with soil at a rate of 2 g per 100 g dry soil. The C, N, P, and S concentrations in the pieces were 417.6, 9.74, 1.20, and 1.19 g kg^−1^, respectively. The SS was regulated by adding or not adding the nutrient solutions (NS_1_, NS_2_, or NS_3_) containing ammonium nitrate, potassium dihydrogen phosphate, and ammonium sulfate (pH = 7). The concentrations of N, P, and S in NS_1_ were 6.42, 1.79, and 0.95 g L^−1^, in NS_2_ were 19.50, 3.52, and 1.90 g L^−1^, and in NS_3_ were 30.72, 9.98, and 4.23 g L^−1^, respectively.

**Table 1:** The amount of substrates added and their stoichiometric ratio.

**Treatment**	**Substrate addition (mg 100 g**^**−1**^ **soil)**				**Stoichiometric ratio**			
	**IH Maize-C**	**Nutrient-N**	**Nutrient-P**	**Nutrient-S**	**C:**	**N:**	**P:**	**S**
CK	0	0	0	0	–	–	–	–
NPS0	250.54	19.49	2.39	2.38	10,000	778	95	95
NPS1	250.54	19.49 + 6.42	2.39 + 1.79	2.38 + 0.95	10,000	1,034	167	133
NPS2	250.54	19.49 + 19.50	2.39 + 3.52	2.38 + 1.90	10,000	1,556	238	171
NPS3	250.54	19.49 + 30.72	2.39 + 9.98	2.38 + 4.23	10,000	2,004	494	264

CK, soil only; NPS0, soil + straw; NPS1–NPS3, soil + straw + incremental nitrogen, phosphorus, and sulfur addition; IH Maize-C, ideal humus amount of straw carbon; C:N:P:S, The stoichiometric ratios of carbon, nitrogen, phosphorus and sulfur in the substrates. The data followed the “+” refers to the amount of extra nutrients added of each treatment.

### 2.3 *In situ* cultivation

Topsoil (0–20 cm) and subsoil (20–40 cm) samples (8 kg each) were collected, sieved at 2 mm, and air-dry. Any visible plant and animal debris and gravel were removed. The SS (NPS1–NPS3) was regulated by adding 1 ml of NS_1_, NS_2_, or NS_3_ to 100 g of dry topsoil on a clean and smooth plastic sheet. The soil moisture was then adjusted to 60% field capacity with distilled water, followed by the addition and mixing of 2 g straw fragments. The mixture was then transferred to nylon mesh bags and sealed (aperture: 0.048 mm, length: 20 cm, and width: 15 cm). The subsoil was treated in the same manner. Nine replicates were maintained for each treatment for both topsoil or subsoil. Three soil pits (length: 1.5 m, width: 0.5 m, depth: 0.4 m) spaced at 0.6 m apart were dug in the field, and the topsoil and subsoil were stored separately. Three replicates of each treatment for subsoil were arranged in two rows (spaced ~20 cm) and vertically placed at 20–40 cm in each pit. Each pit was backfilled with the original subsoil. The same procedure was followed to fill the pits with the replicates of the five treatments for topsoil. Maize (*Xianyu 1483*) was artificially sown in the landfill area (plant spacing: 27 cm and row spacing: 60 cm). All replicates from one pit were collected at 30, 90, and 150 days post-sowing, then brought back to the laboratory with dry ice and stored at −80°C.

### 2.4 Soil basic properties

Part of each replicate was air-dried, and any straw residue was carefully removed using the dry-sieving winnowing method (Kirkby et al., 2014). The sample was then passed through a 0.15-mm sieve. The SOC concentration was determined through potassium dichromate oxidation and ferrous sulfate titration (Kalembasa and Jenkinson, 1973). The soil available P (SAP) concentration was determined through sodium bicarbonate solution leaching colorimetry (Sparks et al., 2020). In another part of the fresh soil sample, any incompletely decomposed straw residues were removed. The sample was examined under a low-power electron microscope to confirm the absence of straw debris. The soil moisture content (SM) was measured using the oven-drying method. The concentration of soil available N [AN, ammonium N (${\text{NH}}_{4}^{+}$-N) and nitrate N (${\text{NO}}_{3}^{-}$-N)] was determined through potassium chloride leaching method (soil-liquid ratio 1:5), followed by analysis with a continuous flow analyzer (FLAStar 5000, Foss, Germany).

### 2.5 Microbial necromass C

MNC was assessed using glucosamine (GlcN) and muramic acid (MurA) as biomarkers. An air-dried sample (1 g) was hydrolyzed at 105°C for 8 h in a hydrolytic tube. Then, 100 μL inositol was added to the tube, mixed, filtered, and dried at 65°C. The residue was dissolved in 20 mL pure water (pH: 6.6–6.8), centrifuged (4,000 rpm, 10 min), and freeze-dried. The freeze-dried residue was dissolved with 5 mL anhydrous methanol, centrifuged, transferred to a 5-mL bottle for derivatization with N-methylglucosamine after freeze-drying. The sample was reacted with 300 μL of derivative reagent, sealed, and swirled for 30 s. After adding 1 mL of acetic anhydride, 1.5 mL of dichloromethane, and 1 mL of hydrochloric acid to the sample in a water bath, the inorganic phase was removed by phase separation, and this process was repeated thrice. The remaining organic phase was dried with N_2_ gas at 45°C, and the residue was dissolved in 300 μL of diluent for gas chromatography analysis (Agilent 6890A, VT-1 column:30 m × 0.25 mm × 0.25 μm). Refer to Chen et al. (2021) for gas flow rate and column heating procedure.

Bacterial NC (BNC) and fungal NC (FNC) were calculated as follows (Appuhn and Joergensen, 2006; Liang et al., 2019):


$$\begin{array}{l} \text{BNC}={\text{C}}_{\text{MurA}}\;\times \;45\\ \text{FNC}=\left({\text{C}}_{\text{GlcN}}/179.17-2\;\times \;{\text{C}}_{\text{MurA}}/251.23\right)\;\times \;179.17\;\times \;9 \end{array}$$

where C_MurA_ and C_GlcN_ are the concentrations of MurA and GlcN, respectively (mg g^−1^), 45 is the conversion coefficient between MurA and BNC, 179.17 and 251.23 are the relative molecular weights of GlcN and MurA, respectively, and 9 is the conversion coefficient between GlcN and FNC (Joergensen, 2018). MNC was estimated as the sum of FNC and BNC (Sradnick et al., 2014).

### 2.6 Plant necromass C

PNC was assessed using lignin phenols (vanillyl (V), syringyl (S), and cinnamyl (C) phenols) as biomarkers (Hedges and Ertel, 1982). Approximately 0.5 g dry soil was mixed with 500 mg CuO, 100 mg ammonium sulfate, 50 mg glucose, 0.4 mL internal standard, and 15 mL NaOH in a Monard reactor. The oxidation products were centrifuged (3,000 rpm, 15 min), acidified (pH 1.8–2.2), and kept in dark for 1 h. After centrifugation, the supernatant was diluted to 100 mL with ethyl acetate, rotary- evaporated (<38°C), and dried with N_2_. The residue was dissolved with 50 μL pyridine and 100 μL N, O-bis (trimethylsilyl) trifluoroacetamide (60°C, 10 min), then analyzed by gas chromatography (Agilent 6890A). PNC is calculated as follows:


$$\begin{array}{l} \text{PNC}=\left({\text{C}}_{\text{V}}/0.33\;+\;{\text{C}}_{\text{S}}/0.9+{\text{C}}_{\text{C}}\right)/\left(0.08\;\times \;1,000\right) \end{array}$$

where C_V_, C_S_, and C_C_ are the C concentrations in V, S, and C, respectively (μg g^−1^). The release coefficients of V, S, and C by CuO oxidation were 0.33, 0.90, and 1.00, respectively (Hautala et al., 1997). The minimum lignin concentration in plant residues of major crops is set to 0.08 (Burgess et al., 2002). One thousand is the conversion coefficient between μg and mg.

### 2.7 Soil microbial traits

The activities of five hydrolases were detected using the 96-well fluorescent plate method according to Saiya-Cork et al. (2002). These included enzymes for C (β-glucosidase, BG; cellobiohydrolase, CBH), N (N-acetyl-β-glucosaminidase, NAG; leucine aminopeptidase, LAP), and P (acid phosphatase, AP) acquisition. Each replicate of a 1-g fresh sample was mixed with the respective substrates and incubated at 25°C for 4 h in the dark (substrates were presented in Supplementary Table S1). The fluorescent plate was reading using a microplate reader to evaluate the enzymatic activities (Synergy H1M, USA, 365 nm excitation and 450 nm emission).

The microbial diversity and composition of were determined through amplicon sequencing. Primers 515F (5′-GTGCCAGCMGCCGCGG-3′) and 907R (5′-CCGTCAATTCMTTTRAGTTT-3′) were used to amplify the V3–V4 region of the 16S rRNA gene. Primers ITS1F (5′-CTTGGTCATTTAGAGGAAGTAA-3′) and ITS2R (5′-GCTGCGTTCTTCATCGATGC-3′) were used to amplify the ITS gene. The 20 μL PCR system contained 4 μL of 5× TransStart FastPfu buffer, 2 μL of 2.5 mM dNTPs, 0.8 μL of each primer, and 0.4 μL TransStart FastPfu DNA polymerase.

PCR products were recovered through 2% agarose gel electrophoresis and purified using the AxyPrep DNA Gel Extraction Kit (Axygen Biosciences, CA, USA). The library was constructed using the NEXTFLEX Rapid DNA-Seq Kit and sequenced using the Miseq platform (Illumina Miseq PE300, USA). Bacteria were classified using the SILVA database (Release 138), while fungi were identified using the UNITE database (Release 8.0) (Quast et al., 2012; Nilsson et al., 2018).

### 2.8 Statistical analysis

Statistical differences in MNC, PNC, BNC, FNC, lignin phenols, SOC, AN, SAP, SM, enzyme activities, α-diversity index and gene abundance (phylum level) among various treatments and soil depths were identified by one-way ANOVA and Duncan's multiple comparisons (*p* < 0.05) in SPSS 19 (IBM, Chicago, IL, USA). The C/V to S/V ratios reflect the intensity of microbial transformation of plant lignin, with a lower ratio indicating a higher transformation intensity (Kögel, 1986). Principal coordinates analysis (PCoA) based on Bray-Curtis distance was employed to assess the dispersion patterns of microbial communities in R (version 4.4.0). Random forest models were used to reveal the influences of soil physical, chemical and microbial traits on soil NC by the “randomForest” and “rfPermute” package according to Archer (2016) in R (version 4.4.0). Finally, a schematic diagram was used to reveal the potential mechanisms and pathways of MNC and PNC formation in topsoil and subsoil. All figures were prepared using SigmaPlot (version 12.5, Systat Software, Chicago, IL, USA).

## 3 Results

### 3.1 Variations of MNC and PNC at different substrate matching levels

**Figure 1:**
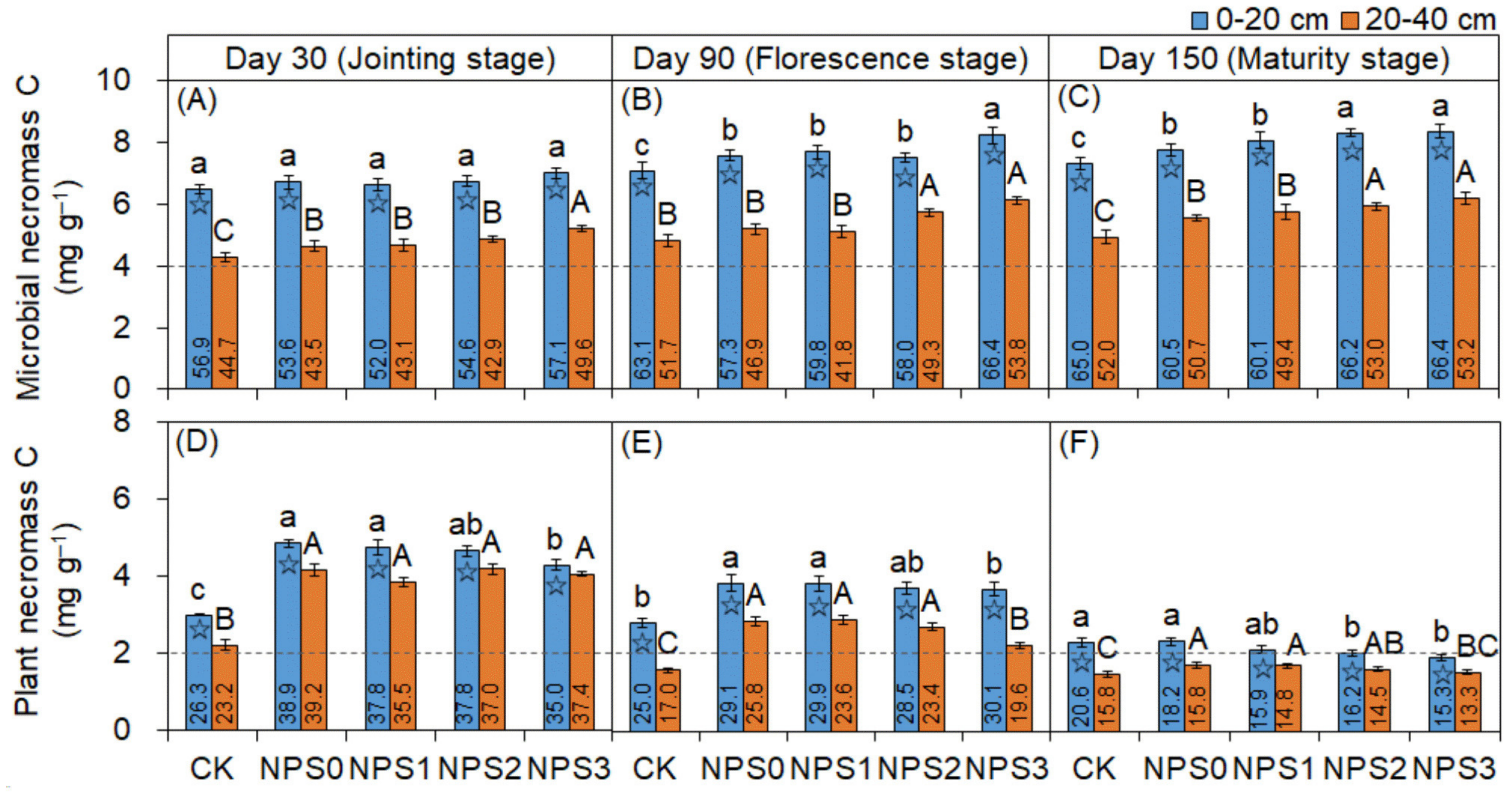
Dynamics of microbial necromass C **(A–C)** and plant necromass C **(D–F)** in topsoil (0–20 cm, blue) and subsoil (20–40 cm, orange) under varied substrate stoichiometry on days 30, 90, and 150. Different lowercase or uppercase letters above bars mean significant differences among treatments in topsoil and subsoil, respectively (*p* < 0.05). Asterisk indicates significant difference between topsoil and subsoil (*p* < 0.05). CK, soil only; NPS0, soil + straw; NPS1–NPS3, soil + straw + incremental nitrogen, phosphorus, and sulfur addition.

MNC and PNC concentrations were influenced by SS (Figure 1). Compared to NPS0, NPS3 increased MNC by 7.6%−8.4% in the topsoil (days 90 and 150) and by 11.8%−18.1% in the subsoil. Concurrently, PNC was reduced by 4.4%−17.5% in the topsoil and by 10.6%−21.7% in the subsoil (days 90 and 150). Whereas, NPS1 did not significantly affect MNC or PNC. Under the same treatments, MNC and PNC concentrations were higher in the topsoil than in the subsoil. Over time, the contribution of MNC to SOC increased from 52.0% to 66.4% in the topsoil and from 42.9% to 53.2% in the subsoil. The contribution of PNC to SOC decreased from 38.9% to 15.3% in the topsoil and from 39.2% to 13.3% in the subsoil.

**Figure 2:**
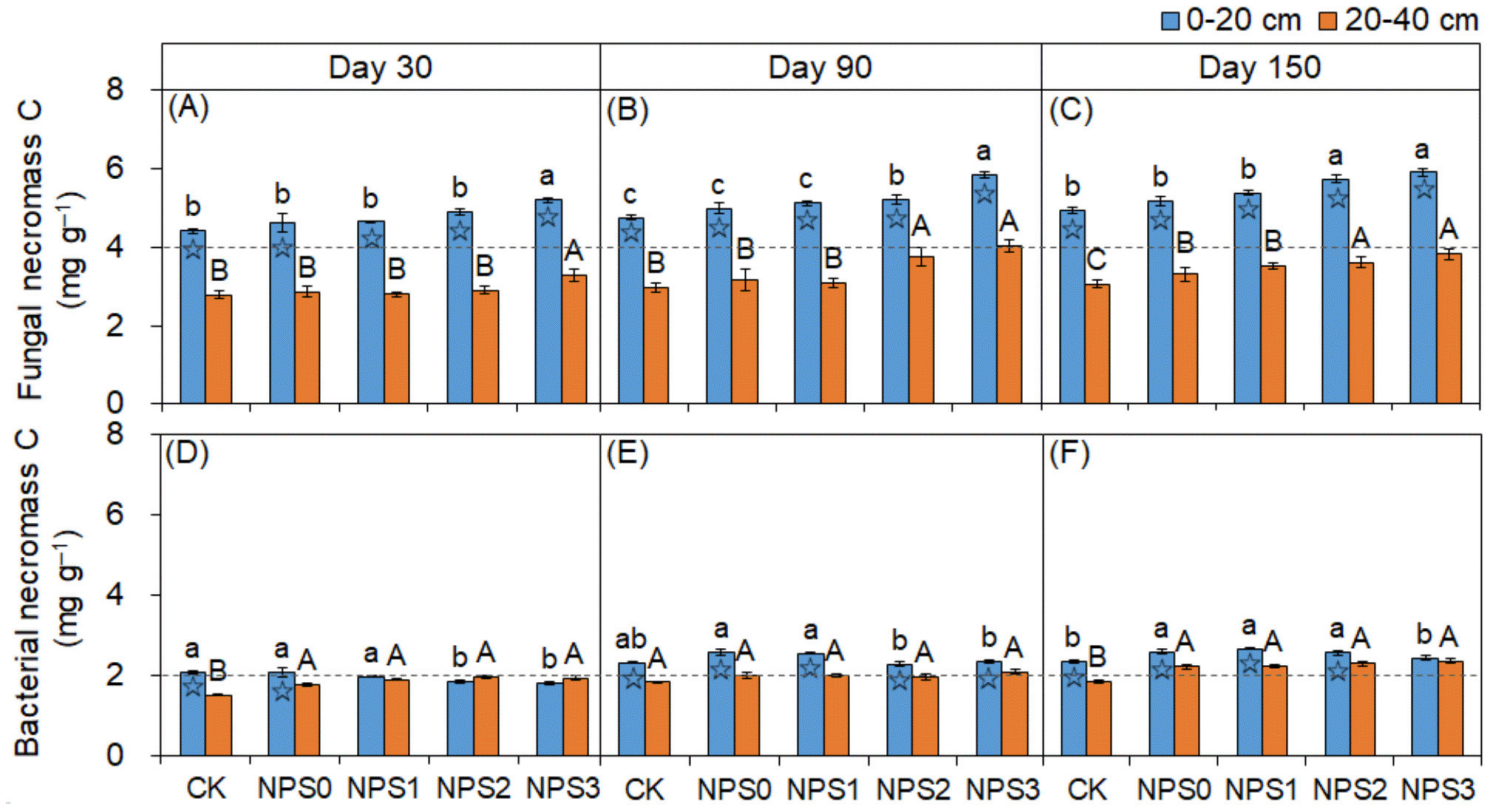
Changes in fungal necromass C **(A–C)** and bacterial necromass C **(D–F)** in topsoil (0–20 cm, blue) and subsoil (20–40 cm, orange) under different substrate stoichiometries on days 30, 90, and 150. Different lowercase or uppercase letters mean significant differences among treatments in topsoil and subsoil, respectively (*p* < 0.05). Asterisks denote significant differences between topsoil and subsoil (*p* < 0.05). CK, soil only; NPS0, soil + straw; NPS1–NPS3, soil + straw + incremental nitrogen, phosphorus, and sulfur addition.

**Figure 3:**
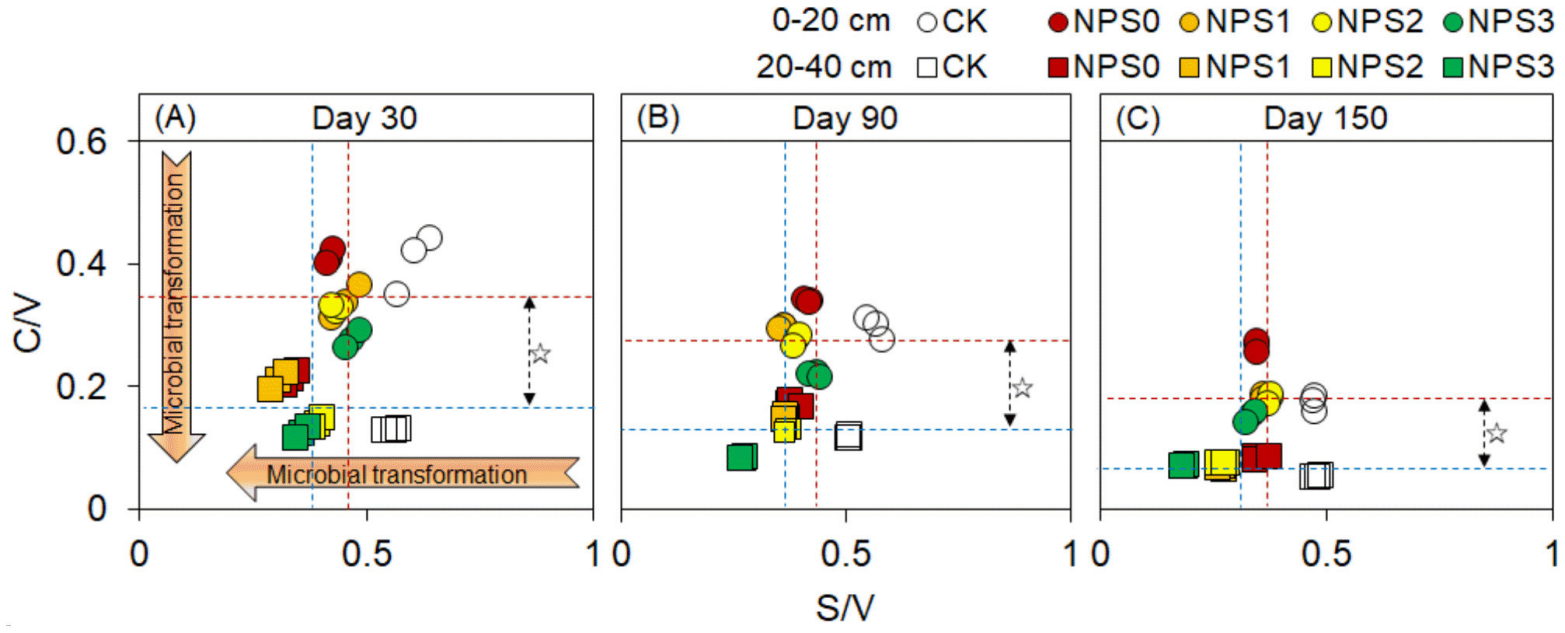
**(A–C)** Ratios of cinnamyl to vanillyl phenol (C/V) vs syringyl to vanillyl (S/V) for lignin phenols in topsoil (0–20 cm, circle) and subsoil (20–40 cm, square)under different substrate stoichiometric ratios on days 30, 90, and 150. Red and blue dashed lines represent the mean values for topsoil and subsoil, respectively (*n* = 3). Asterisks indicate significant differences between topsoil and subsoil (*p* < 0.05). CK, soil only; NPS0, soil + straw; NPS1–NPS3, soil + straw + incremental nitrogen, phosphorus, and sulfur addition.

FNC was more responsive to changes in SS than BNC (Figure 2). Compared with NPS0, NPS3 increased FNC by an average of 14.8% in both topsoil and 20.8% in the subsoil. In contrast, NPS3 reduced BNC in the topsoil by 6.0%−13.6%, with no change in the subsoil. The FNC and BNC concentrations in the topsoil were 1.4–1.7 times and 0.9–1.3 times those in the subsoil, respectively. The microbial transformation intensity of phenols gradually increased from NPS0 to NPS3, with the subsoil showing higher intensity than the topsoil (Figure 3).

### 3.2 Response of soil basic properties to substrate stoichiometry

**Figure 4:**
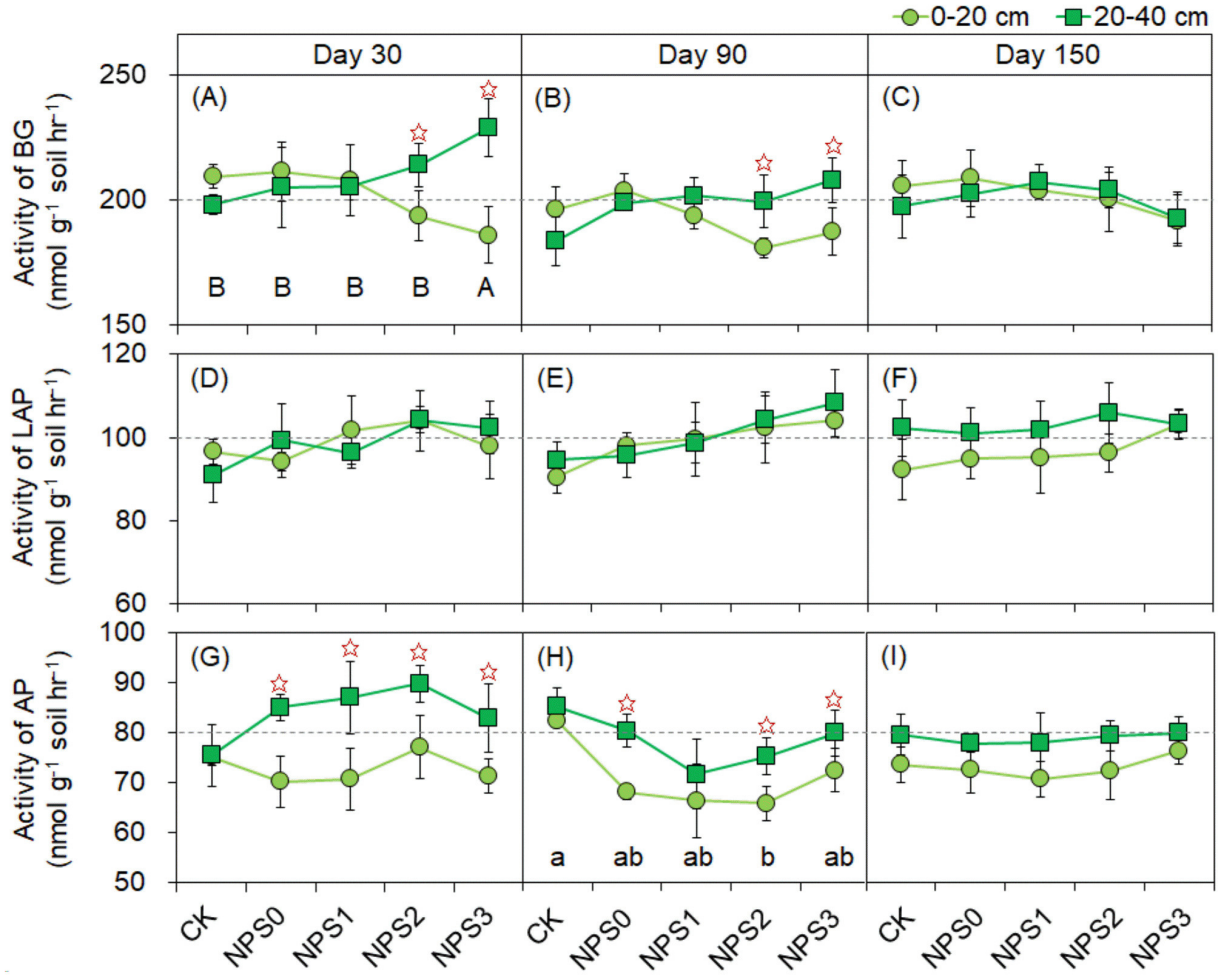
Activities of enzymes involved in C **(A–C)**, N **(D–F)**, and P **(G–I)** acquisition under different substrate stoichiometries at three sampling times. Different lowercase or uppercase letters mean significant differences among treatments in topsoil and subsoil, respectively (*p* < 0.05). Asterisks indicate significant differences between topsoil and subsoil (*p* < 0.05). BG, β-glucosidase; LAP, L-leucine aminopeptidase; AP, acid phosphatase; CK, soil only; NPS0, soil + straw; NPS1–NPS3, soil + straw + incremental nitrogen, phosphorus, and sulfur addition.

Compared with NPS0, NPS1–NPS3 increased the SM of the subsoil by 8.7%−23.6% on day 30, while no change was observed in the topsoil (Supplementary Figure S2). The concentrations of AN and SAP increased from NPS1 to NPS3. For instance, the SAP concentration in the topsoil increased from 18.2 mg kg^−1^ (NPS0) to 32.7 mg kg^−1^ (NPS3). The activities of BG (NPS3) and AP (NPS0–NPS3) were higher in the subsoil than in the topsoil at days 30 and 90 (Figure 4). Compared with NPS0, only NPS3 decreased the Ace of bacteria by 10.6% in the subsoil on day 30, with no change was observed in other treatments or soil layers (Supplementary Figures S4A–C). No change was observed in the Ace of fungi among NPS0–NPS3 (Supplementary Figures S4G–I). PCoA analysis showed that the incremental addition of nutrient substrates increased the dispersion degree of both bacterial and fungal communities compared with NPS0 (Supplementary Figure S5).

**Figure 5:**
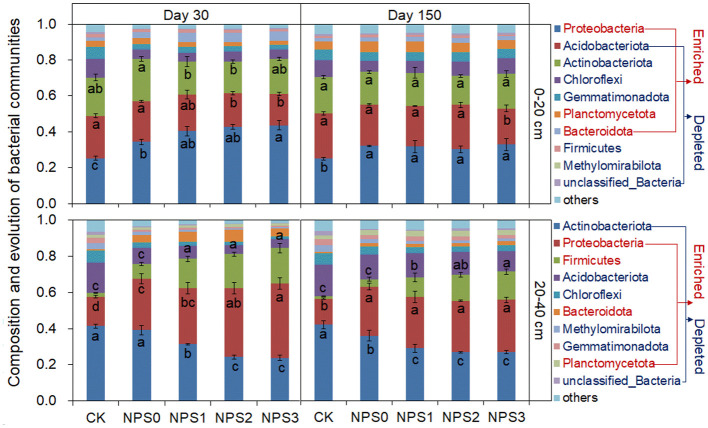
Bacterial community composition on days 30 and 150 under varying substrate stoichiometries in topsoil and subsoil. Different letters indicate significant differences among treatments (*p* < 0.05). CK, soil only; NPS0, soil + straw; NPS1–NPS3, soil + straw + incremental nitrogen, phosphorus, and sulfur addition.

In the topsoil, *Proteobacteria, Acidobacteriota*, and *Actinobacteriota* predominanted, accounting for 34%−44%, 17%−22%, and 18%−24% of the bacterial community, respectively. In the subsoil, the dominant bacteria were *Actinobacteriota, Proteobacteria*, and *Firmicutes*, accounting for 24%−42%, 14%−41%, and 8%−19%, respectively (Figure 5). Compared to NPS0, NPS1–NPS3 enriched the abundances of *Proteobacteria, Planctomycetota*, and *Bacteroidota* in both topsoil and subsoil, while the *Firmicutes* abundance increased only in the subsoil. Fungal compositions were similar between topsoil and subsoil. NPS1 increased *Mortierellomycota* abundance by 61%, but had no effect on Ascomycota. NPS3 increased *Ascomycota* and *Mortierellomycota* abundances by 23% and −24.7%, respectively (Supplementary Figure S6).

### 3.3 Control factors over MNC and PNC formation

**Figure 6:**
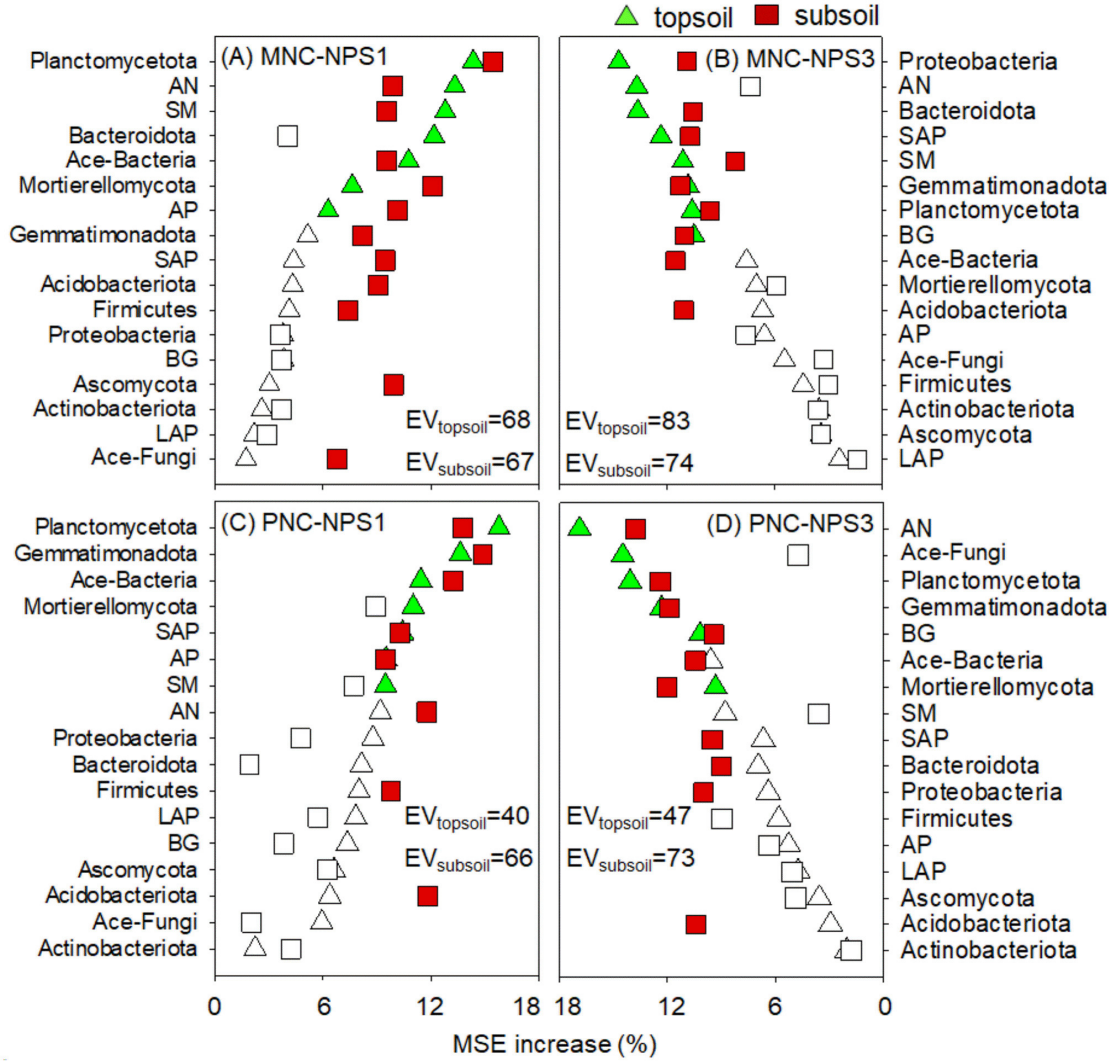
Relative influence of soil variables on microbial and plant necromass C (MNC, PNC) under oligotrophic **(A, C)** and copiotrophic conditions **(B, D)** in topsoil and subsoil by the percentage increase of the mean squared error (MSE). Significant variables are highlighted in green and red for topsoil and subsoil, respectively (*p* < 0.05). SM, soil moisture; AN, soil available nitrogen; SAP, soil available phosphorus; BG, β-glucosidase; AP, acid phosphatase; EV, total explained variance; NPS1, substrate stoichiometry to match fungal needs; NPS3, substrate stoichiometry to match bacterial needs.

Seventeen variables were used to reflect the impact of soil physical, chemical and biological parameters on NC (Figure 6). For NPS1, *Planctomycetota*, AN, SM, Ace-Bacteria, and AP were the primary factors regulating MNC changes, with the effects of *Mortierellomycota, Firmicutes*, and SAP on MNC in the subsoil being more pronounced. *Planctomycetota*, Ace-Bacteria, SAP, AP, and SM were the most important factors regulating PNC changes, with the effects of AN and *Firmicutes* being enhanced in the subsoil. For NPS3, MNC changes were more reliant on *Proteobacteria*, AN, SAP, SM, and BG, with the effects of Ace-Bacteria on MNC in the subsoil being more pronounced. PNC changes were predominantly influenced by *Planctomycetota*, AN, Ace-Fungi, BG, and *Mortierellomycota*, with SAP's influence being more pronounced in the subsoil.

**Figure 7:**
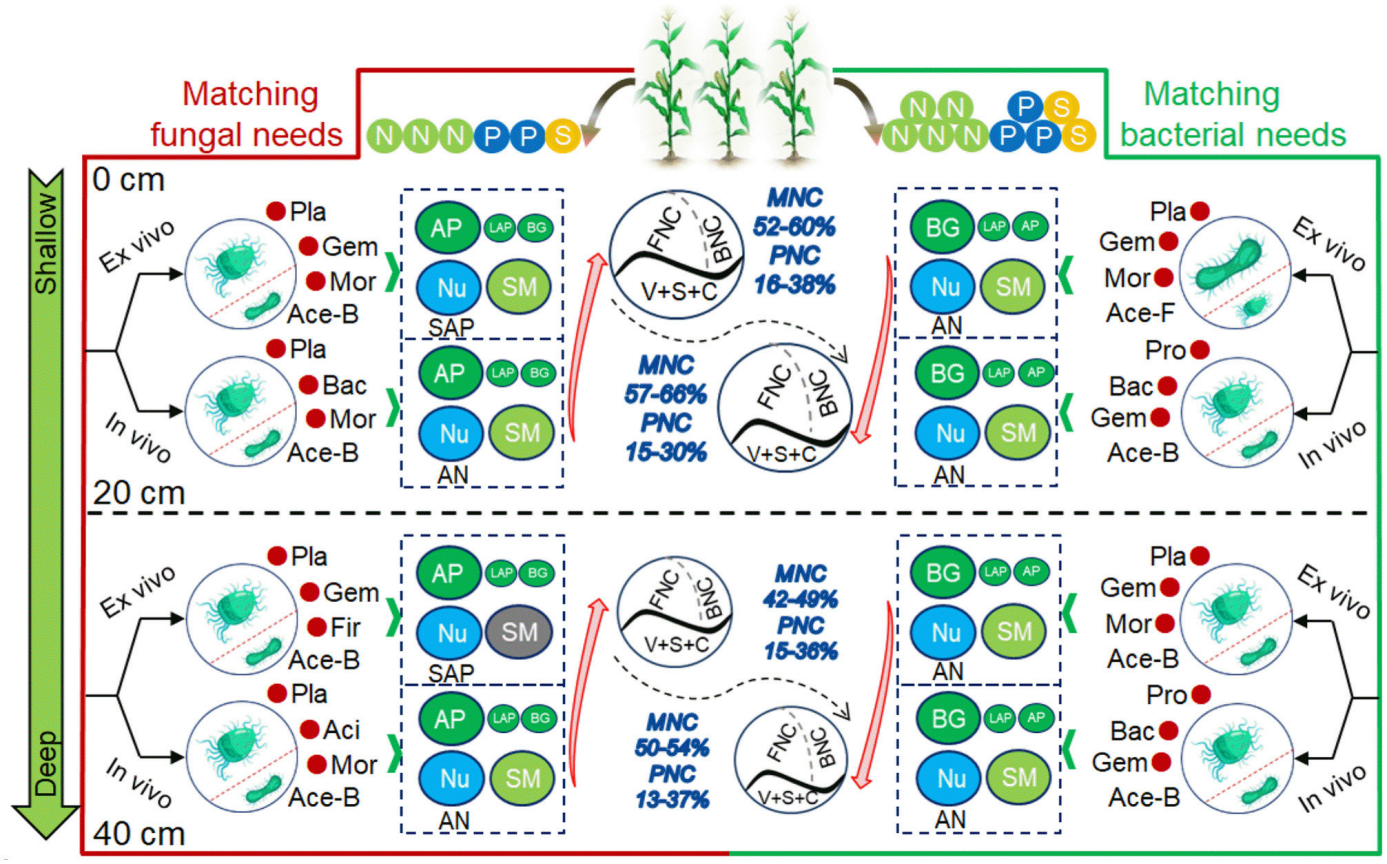
Schematic diagram of MNC and PNC formation under substrate stoichiometric matching (oligotrophic to copiotrophic) in topsoil and subsoil. MNC, microbial necromass C; PNC, plant necromass C; FNC, fungal necromass C; BNC, bacterial necromass C; V, S and C, vanillyl, syringyl, and cinnamyl phenols; Nu, nutrient availability; SM, soil moisture; AN, soil available nitrogen; SAP, soil available phosphorus; BG, β-glucosidase; LAP, L-leucine aminopeptidase; AP, acid phosphatase; Ace-B/F, Acetate of bacteria/fungi; Pla, Planctomycetota; Bac, Bacteroidota; Act, Actinobacteriota; Gem, Gemmatimonadota; Aci, Acidobacteriota; Asc, Ascomycota; Mor, Mortierellomycota; Glo, Glomeromycota.

The schematics of the evolution of NC were constructed by key factors under SS_O_ and SS_C_ (Figure 7). Under NPS1, dominant bacteria (e.g., *Planctomycetota*), AP, and SM controlled NC changes, with MNC and PNC being more affected by AN and SAP, respectively. *Firmicutes* significantly impacted NC in the subsoil. Under NPS3, a broader range of bacteria, including *Proteobacteria*, were mobilized, with BG and AN playing important roles in NC changes. Fungi and their richness controlled more in the variation of PNC in topsoil.

## 4 Discussion

### 4.1 Changes in MNC and PNC in response to substrate stoichiometry

The accumulation of MNC positively responded to the integration of straw with N, P, and S (Figures 1A–C), which was consistent with the hypothesis I. Generally, C-rich but nutrient-poor substrates (NPS0) limit microbial anabolism, while C- and nutrient-rich substrates (NPS3) promote anabolic processes by satisfying microbial stoichiometric demands (Heuck et al., 2015). Our previous study also established a positive correlation between microbial C assimilation and nutrient availability (Wu et al., 2023). Optimizing the SS to meet the needs from fungi to bacteria promotes microbial proliferation and accelerates growth cycles, particularly for fungi, thus enhancing MNC accumulation (Liang et al., 2007; Tian et al., 2024). Consistent with the previous findings, this study also found that the MNC concentration in the topsoil was higher than that in the subsoil, primarily due to the frequent input, and decomposition of plant-derived C (Cotrufo et al., 2013; Zhou et al., 2023). The 15% increase of MNC in the subsoil (vs. 8% in the topsoil) highlighted SS's role in overcoming the nutrient limitations in less disturbed subsoil (Zhang et al., 2020). Thus, adjusting SS according to bacterial metabolic needs promoted MNC accumulation in the short-term field trials.

PNC dynamics further underscored this mechanism: its decline under NPS3 (Figures 1D–F) revealed a SS-driven shift from *ex vivo* modification to *in vivo* turnover. Upon straw incorporation, microbes and enzymes preferentially decomposed labile C, leaving behind *ex vivo*-modified recalcitrant C and driving PNC accumulation (Liang et al., 2017). However, nutrient supplementation enabled microbes to reuse the remaining C via faster growth cycles, and the C/V and S/V ratios confirmed the gradual microbial transformation of PNC (Figure 3) and the “entombing effect” of MNC (Fang et al., 2018; Tian et al., 2024). The topsoil's lower PNC transformation intensity compared to the subsoil could be linked to the continuous input of plant materials, leading to rapid C component update and reduced metabolic extents (Kögel, 1986; Chen et al., 2021). Moreover, the higher water availability in the subsoil may promote microbial transformation (Matthews et al., 2023). Therefore, nutrient supplementation induced PNC digestion and its potential transfer to MNC.

The dominance of FNC over BNC (Figure 2) indicated the distinct responses of oligotrophs and copiotrophs to SS variations. The oligotrophic nature of fungi meant that improved substrate accessibility both shortened their life cycles and mediated community selection, ultimately promoting FNC accumulation (Fang et al., 2018; Tian et al., 2024). While, bacterial necromass is preferentially dissolved duo to structural weakness, which precisely explains that the contribution of FNC to MNC is greater than that of BNC (Nakas and Klein, 1979). The distribution difference of FNC between topsoil and subsoil was more pronounced, as aerobic topsoil conditions further amplified its accumulation (Moritz et al., 2009). Therefore, the variations of FNC and BNC were influenced by microbial life strategies and environmental conditions, with FNC contributing more to MNC.

### 4.2 Evolutions of biochemical properties in response to substrate stoichiometry

The regulation of SS altered the biochemical properties of both topsoil and subsoil in the short term, highlighting the tightly interplay between soil nutrient dynamics and microbial responses. Soil nutrient capacity especially SAP increase, from NPS0 to NPS3 (Supplementary Table S2), was driven not only by direct P amendments but also by the integration of straw, which acts as a conduit for P activation (Wu et al., 2022). Moreover, straw incorporation activated a synergistic action of *Pseudomonas* and phosphatase, substantially improving soil P availability (Hu et al., 2018). The subsoil showed enhanced nutrient buildup relative to the topsoil, resulting from its native nutrient deficiency and oxygen-limited conditions conducive to nutrient retention (Chen et al., 2020). Such conditions, while suppressing aerobic processes, unexpectedly intensify substrate amendment effects, as indirectly evidenced by the sensitive response of microbial community's dispersion degree to substrate addition in the subsoil (Supplementary Figure S5). Additionally, water availability regulated NC formation by enhancing subsoil microbial activity and substrate diffusion, thereby supporting microbial growth and subsequent NC production (Matthews et al., 2023).

The hydrolases involved in *ex vivo* modification were significantly influenced by the SS (Liang et al., 2017; Jiang et al., 2019). Our results revealed that optimized SS preferentially stimulated BG and AP activities in the subsoil, exceeding topsoil enzyme levels during early decomposition stages (Figure 4). The enhanced enzymatic activity demonstrated significantly increased substrate decomposition potential and nutrient cycling efficiency in the subsoil, challenging conventional views of subsoil inactivity while highlighting its sensitivity to nutrient amendments. Moreover, the narrow C:N:P acquisition enzyme ratios were observed among different treatments (Supplementary Table S3). Such metabolic consistency reflected microbial adaptive strategies that maintain a balanced enzymatic profile to engage in stoichiometric metabolism, ensuring integrated acquisition and utilization of substrate nutrients (Sinsabaugh et al., 2009; Luo et al., 2017). The declining C:N and C:P enzyme activity ratios observed in the topsoil from NPS0 to NPS3 likely resulted from oxygen-replete conditions and enhanced substrate availability, which promoted more efficient C assimilation over nutrient mining (Fang et al., 2018; Chen et al., 2018). This, in turn, promoted the production of nutrient-acquiring enzymes, effectively mitigating microbial biomass stoichiometric imbalances (especially for eutrophic environment). Therefore, microbial communities adjusted their enzymatic strategies in response to SS, with BG and AP serving as key drivers of nutrient cycling.

The primarily microbial taxa that dominated the *in vivo* turnover and promoted microbial biomass assimilation process were significantly influenced by SS (Figure 5 and Supplementary Figure S5). Eutrophic bacteria (e.g., *Proteobacteria* and *Planctomycetota*), which actively participate in organic matter digestion and assimilation, demonstrated effective mobilization across soil horizons (Fang et al., 2018; Kim et al., 2021). The predominance of *Proteobacteria* in driving both microbial proliferation and necromass accumulation under SS_C_ conditions stems from their preferential substrate utilization and central role in microbial assimilation pathways (Kim et al., 2021; Shi et al., 2023). *Firmicutes*, typically deep-seated bacteria, were selectively mobilized in the subsoil, demonstrating notable adaptability for C decomposition and recycling under oxygen- and nutrient-limited conditions (Zhang et al., 2021). Fungal taxa, *Ascomycota* and *Mortierellomycota*, established dominance as primary decomposers across soil layers, exhibiting enzymatic capabilities for labile substrate degradation including sugars and cellulose (Pei et al., 2019). Notably, *Mortierellomycota*, a nutrient-sensitive fungal phylum, demonstrated preferential activity in SS_O_ conditions, as evidenced by its relatively high abundance under NPS1 (Supplementary Figure S6). Substrate amendments substantially changed *in vivo* microbial turnover through selective mobilization of key taxa (Proteobacteria, Planctomycetota, and Mortierellomycota), which subsequently drived microbial assimilation processes.

### 4.3 Formation of necromass C regulated by substrate stoichiometry

The dynamics of MNC and PNC were influenced by varying factors from SS_O_ to SS_C_ in our short-term experiment (Figure 6). While P availability selectively affected PNC formation under SS_O_, N availability universally dominated necromass regulation, consistent with its known multifunctional control over C catabolism and anabolism (Vu et al., 2022). Under SS_O_, the catabolism of C source faces with nutrient limitations, particularly P limitation due to its strong adsorption capacity and rapid fixation in soil. To meet microbial substrate decomposition requirements, nutrient acquisition enzymes such as AP are required to release P from plant and soil (Jiang et al., 2019). This explained the marked regulatory effect of AP activity on NC changes under NPS1. Conversely, under SS_C_, BG-mediated processes became the primary driver of NC change, reflecting C anabolism and recycling in bacterial-favoring environments that preferentially rely on C-acquiring enzymatic pathways. As discussed in Section 4.2, the chemotrophic preferences indicated that *Firmicutes* significantly impacted NC under SS_O_ (especially in the subsoil), while *Proteobacteria* played an enhanced role under SS_C_. In addition to bacteria, fungi also control over NC variations (Figures 6A, D), particularly through *Mortierellomycota*-mediated mobilization of bioavailable P to maintain microbial stoichiometric balance (Zhang et al., 2011). Thus, SS regulated NC dynamics by integrating the key roles of nutrient availability, enzymatic activities, and microbial strategies.

SS_C_ significantly promoted the continuous accumulation of MNC (Figure 7). In SS_O_-treated topsoil, dominant bacteria taxa cooperated with specialized fungal decomposers to employed P-acquiring enzymes to process and assimilate C sources. The availability of water and nutrient (especially N) showed significant positive correlations with this microbial process, underscoring the critical role of nutrient adequacy in regulating microbial metabolic activity. Under SS_C_, both the rate of MNC accumulation and its contribution to SOC showed gradually enhancement, predominantly driven by *Proteobacteria* and BG, alongside an increased incorporation of FNC. Generally, elevated nutrient availability preferentially stimulated copiotrophic bacterial activity, intensifying their contribution to MNC formation through BG-mediated C acquisition pathways, while simultaneously releasing BNC and FNC via turnover processes. The selective suppression of oligotrophic fungal taxa under eutrophic environments resulted in enhanced accumulation of FNC (Jiang et al., 2019; Megyes et al., 2021). Moreover, the structural stabilization of FNC reduces its bioavailability for microbial reuse, thus promoting its contribution to MNC over BNC (Nakas and Klein, 1979). The influence of SS_C_ on MNC in the subsoil was similar to that in the topsoil, though with a greater accumulation intensity in the subsoil. This disparity may be attributed to the inherent characteristics of the subsoil, as discussed in Section 4.1. In summary, SS meeting bacterial metabolic needs promoted MNC accumulation, which were mainly associated with dominant bacteria, N and P availability, and C-acquiring enzymes.

SS_C_ also enhanced the consumption and transformation of PNC in both topsoil and subsoil. Under SS_O_, dominant bacteria taxa cooperated with certain fungal group to primarily decomposed labile substrates through AP-mediated P acquisition, while recalcitrant macromolecules (e.g., lignin phenols) underwent temporal stabilization, directly contributing to PNC. The availability of P demonstrated a significant positive correlation with this process, highlighting its critical role in facilitating microbial decomposition activities (Wu et al., 2023). Under SS_C_, both PNC concentration and its contribution to SOC showed gradually declines, a pattern closely associated with N availability, BG activity, and *Mortierellomycota* abundance. In such environments, enhanced microbial assimilation of substrates leads to the transformation of plant-derived C to MNC via *in vivo* turnover. Therefore, the requirement for C-acquiring enzymes becomes more critical to convert partly decomposed plant material. Following depletion of labile plant substrates, previously sequestered PNC may undergo progressive microbial reactivation and catabolism (Liang et al., 2017; Chen et al., 2021). Meanwhile, certain fungal taxa, notably *Mortierellomycota*, may regulate bioavailable nutrient fluxes through dormancy or competition, thus influencing the dynamics of PNC (Zhang et al., 2011). The shift from SS_O_ to SS_C_ attenuated P control over PNC while amplifying N regulation, reflecting SS-driven microbial resource allocation strategies. That is, P plays a central role in microbial catabolism under SS_O_, while microbial C assimilation is more reliant on N availability under SS_C_ (Luo et al., 2017; Jiang et al., 2019; Vu et al., 2022). Therefore, SS meeting bacterial demands enhances the transformation of PNC by primarily regulating N availability, dominant fungi and bacteria, and C-acquiring enzymes.

## 5 Conclusion

This short-term study revealed that aligning substrate stoichiometry (SS) with microbial metabolic requirements induced adaptive shifts in microbial communities, enzymatic activities, and nutrient availability, thereby influencing the formation and transformation of MNC and PNC. Specifically, SS meeting bacterial requirements (SS_B_) enhanced MNC accumulation and accelerated PNC digestion, especially in the subsoil, compared to SS preferred by fungi (SS_F_). This enhancement is attributed to the stoichiometric supply of C, N, P, and S, which meets the metabolic demands of bacteria and supports microbial anabolism. Notably, the preferential release of FNC over BNC emerged as a key driver of MNC accumulation. Consequently, the efficiency and stability of MNC accumulation, particularly FNC, deserve further study to optimize SOC sequestration strategies. The formation of necromass C was predominantly controlled by certain bacteria taxa, such as *Planctomycetota* and *Proteobacteria*, while certain fungi, like *Mortierellomycota*, regulated the release or retention of soil nutrients. Our results highlighted the critical role of P-acquiring enzymes and P availability in microbial catabolism under SS_F_, and the role of C-acquiring enzymes in microbial anabolism under SS_B_. These findings emphasize the importance of aligning straw and nutrient inputs with bacterial metabolic demands to enhance MNC entombment and highlight the potential of subsoil MNC accumulation for C sequestration in the short term.

## Data Availability

The data that support the findings of this study are openly available at https://doi.org/10.5281/zenodo.16310622.

## References

[B1] Appuhn A. Joergensen R. G. (2006). Microbial colonisation of roots as a function of plant species. Soil Biol. Biochem. 38, 1040–1051. 10.1016/j.soilbio.2005.09.002

[B2] Archer E. (2016). rfPermute, Estimate Permutation p-Values for Random. Forest Importance Metrics. R package version 1.5.2. Available online at: https://CRAN.R-project.org/package=rfPermute

[B3] Burgess M. S. Mehuys G. R. Madramootoo C. A. (2002). Decomposition of grain-corn residues (*Zea mays* L.): a litterbag study under three tillage systems. Can. J. Soil Sci. 82, 127–138. 10.4141/S01-013

[B4] Chen H. Li D. J. Zhao J. Zhang W. Xiao K. C. Wang K. L. (2018). Nitrogen addition aggravates microbial carbon limitation: evidence from ecoenzymatic stoichiometry. Geoderma 329, 61–64. 10.1016/j.geoderma.2018.05.019

[B5] Chen R. Senbayram M. Blagodatsky S. Myachina O. Dittert K. Lin X.. (2014). Soil C and N availability determine the priming effect: microbial N mining and stoichiometric decomposition theories. Glob. Change Biol. 20, 2356–2367. 10.1111/gcb.12475 24273056

[B6] Chen X. Hu Y. Xia Y. Zheng S. Ma C. Rui Y.. (2021). Contrasting pathways of carbon sequestration in paddy and upland soils. Glob. Change Biol. 27, 2478–2490. 10.1111/gcb.15595 33713528 PMC8251767

[B7] Chen X. Xia Y. Rui Y. Ning Z. Hu Y. Tang H.. (2020). Microbial carbon use efficiency, biomass turnover, and necromass accumulation in paddy soil depending on fertilization. Agr. Ecosyst. Environ. 292:106816. 10.1016/j.agee.2020.106816

[B8] Chen Z. Ma J. Wang F. Ma J. Zou P. Sun W.. (2025). Long-term phosphorus addition enhances the contributions of plant lignin and microbial necromass to soil organic carbon in a rice–wheat rotation. Appl. Soil Ecol. 209:106010. 10.1016/j.apsoil.2025.106010

[B9] Cotrufo M. F. Wallenstein M. D. Boot C. M. Denef K. Paul E. (2013). The Microbial Efficiency Matrix Stabilization (MEMS) framework integrates plant litter decomposition with soil organic matter stabilization: do labile plant inputs form stable soil organic matter? Glob. Change Biol. 19, 988–995. 10.1111/gcb.12113 23504877

[B10] Fang Y. Y. Nazaries L. Singh B. K. Singh B. P. (2018). Microbial mechanisms of carbon priming effects revealed during the interaction of crop residue and nutrient inputs in contrasting soils. Glob. Change Biol. 24, 2775–2790. 10.1111/gcb.14154 29603502

[B11] Hautala K. Peuravuori J. Pihlaja K. (1997). Estimation of origin of lignin in humic DOM by CuO oxidation. Chemosphere 35, 809–817. 10.1016/S0045-6535(97)00201-4

[B12] Hedges J. I. Ertel J. R. (1982). Characterization of lignin by gas capillary chromatography of cupric oxide oxidation products. Anal. Chem. 54, 174–178. 10.1021/ac00239a00717475307

[B13] Heuck C. Weig A. Spohn M. (2015). Soil microbial biomass C:N:P stoichiometry and microbial use of organic phosphorus. Soil Biol. Biochem. 85, 119–129. 10.1016/j.soilbio.2015.02.029

[B14] Hu P. Zhang W. Kuzyakov Y. Xiao L. Xiao D. Xu L. in.. (2023). Linking bacterial life strategies with soil organic matter accrual by karst vegetation restoration. Soil Biol. Biochem. 177:108925. 10.1016/j.soilbio.2022.108925

[B15] Hu Y. Xia Y. Sun Q. Liu K. Chen X. Ge T.. (2018). Effects of long-term fertilization on phoD-harboring bacterial community in Karst soils. Sci. Total Environ. 628–629, 53–63. 10.1016/j.scitotenv.2018.01.314 29428860

[B16] Jiang Y. L. Lei Y. B. Qin W. Korpelainen H. Li C. Y. (2019). Revealing microbial processes and nutrient limitation in soil through ecoenzymatic stoichiometry and glomalin-related soil proteins in a retreating glacier forefield. Geoderma 338, 313–324. 10.1016/j.geoderma.2018.12.023

[B17] Joergensen R. G. (2018). Amino sugars as specific indices for fungal and bacterial residues in soil. Biol. Fert. Soils 54, 559–568. 10.1007/s00374-018-1288-3

[B18] Kalembasa S. J. Jenkinson D. S. (1973). A comparative study of titrimetric and gravimetric methods for the determination of organic carbon in soil. J. Sci. Food Agric. 24, 1085–1090. 10.1002/jsfa.2740240910

[B19] Kim H. S. Lee S. H. Jo Y. H. Finneran K. T. Kwon M. J. (2021). Diversity and composition of soil *Acidobacteria* and *Proteobacteria* communities as a bacterial indicator of past land-use change from forest to farmland. Sci. Total Environ. 797:148944. 10.1016/j.scitotenv.2021.148944 34298360

[B20] Kirkby C. A. Richardson A. E. Wade L. J. Batten G. D. Blanchard C. Kirkegaard J. A. (2013). Carbon-nutrient stoichiometry to increase soil carbon sequestration. Soil Biol. Biochem. 60, 77–86. 10.1016/j.soilbio.2013.01.011

[B21] Kirkby C. A. Richardson A. E. Wade L. J. Passioura J. B. Batten G. D. Blanchard C.. (2014). Nutrient availability limits carbon sequestration in arable soils. Soil Biol. Biochem. 68, 402–409. 10.1016/j.soilbio.2013.09.032

[B22] Kögel I. (1986). Estimation and decomposition pattern of the lignin component in forest humus layers. Soil Biol. Biochem. 18, 589–594. 10.1016/0038-0717(86)90080-5

[B23] Lavallee J. M. Soong J. L. Cotrufo M. F. (2019). Conceptualizing soil organic matter into particulate and mineral-associated forms to address global change in the 21 stcentury. Glob. Change Biol. 26, 261–273. 10.1111/gcb.14859 31587451

[B24] Li J. Zhang X. Luo J. Lindsey S. Zhou F. Xie H.. (2020). Differential accumulation of microbial necromass and plant lignin in synthetic versus organic fertilizer-amended soil. Soil Biol. Biochem. 149:107967. 10.1016/j.soilbio.2020.107967

[B25] Li J. Zhao J. Liao X. Hu P. Wang W. Ling Q.. (2024). Pathways of soil organic carbon accumulation are related to microbial life history strategies in fertilized agroecosystems. Sci. Total Environ. 927:172191. 10.1016/j.scitotenv.2024.172191 38588738

[B26] Liang C. Amelung W. Lehmann J. Kästner M. (2019). Quantitative assessment of microbial necromass contribution to soil organic matter. Glob. Change Biol. 25, 3578–3590. 10.1111/gcb.14781 31365780

[B27] Liang C. Schimel J. P. Jastrow J. D. (2017). The importance of anabolism in microbial control over soil carbon storage. Nat. Microbiol 2:17105. 10.1038/nmicrobiol.2017.105 28741607

[B28] Liang C. Zhang X. Rubert K. F. Balser T. C. (2007). Effect of plant materials on microbial transformation of amino sugars in three soil microcosms. Biol. Fert. Soils 43, 631–639. 10.1007/s00374-006-0142-1

[B29] Liu H. Wang J. Sun X. McLaughlin N. Jia S. Liang A.. (2023). The driving mechanism of soil organic carbon biodegradability in the black soil region of Northeast China. Sci. Total Environ. 884:163835. 10.1016/j.scitotenv.2023.163835 37137375

[B30] Luo L. Meng H. Gu J. D. (2017). Microbial extracellular enzymes in biogeochemical cycling of ecosystems. J. Environ. Manag. 197, 539–549. 10.1016/j.jenvman.2017.04.023 28419976

[B31] Matthews K. Facelli J. Cavagnaro T. (2023). Response of soil microbial community structure, carbon and nitrogen cycling to drying and rewetting. Appl. Soil Ecol. 192:105099. 10.1016/j.apsoil.2023.10509927486444

[B32] Megyes M. Borsodi A. K. Árendás T. Márialigeti K. (2021). Variations in the diversity of soil bacterial and archaeal communities in response to different long-term fertilization regimes in maize fields. Appl. Soil Ecol. 168:104120. 10.1016/j.apsoil.2021.104120

[B33] Moritz L. K. Liang C. Wagai R. Kitayama K. Balser T. C. (2009). Vertical distribution and pools of microbial residues in tropical forest soils formed from distinct parent materials. Biogeochemistry 92, 83–94. 10.1007/s10533-008-9264-x

[B34] Nakas J. P. Klein D. A. (1979). Decomposition of microbial cell components in a semiarid grassland soil. Appl. Environ Microb. 38, 454–460. 10.1128/aem.38.3.454-460.1979 16345434 PMC243516

[B35] Nilsson R. H. Larsson K. H. Taylor A. F. S. Johan B. P. Jeppesen T. S. Schigel D.. (2018). The UNITE database for molecular identification of fungi: handling dark taxa and parallel taxonomic classifications. Nucleic Acids Res. 47, 259–264. 10.1093/nar/gky1022 30371820 PMC6324048

[B36] Pei G. Guo J. Wang Q. Kang Z. (2019). Comparative analysis of protein kinases and associated domains between *Ascomycota* and *Basidiomycota*. J. Integr. Agric. 18, 96–107. 10.1016/S2095-3119(18)62022-2

[B37] Qi J. Yao X. Duan M. Huang X. Fan M. Yang Y.. (2023). Effects of contrasting tillage managements on the vertical distribution of plant- and microbial-derived carbon in rice paddy. Sci. Total Environ. 892:164348. 10.1016/j.scitotenv.2023.164348 37236452

[B38] Quast C. Pruesse E. Yilmaz P. Gerken J. Glckner F. O. (2012). The SILVA ribosomal RNA gene database project: improved data processing and web-based tools. Nucleic Acids Res. 41, 590–596. 10.1093/nar/gks1219 23193283 PMC3531112

[B39] Saiya-Cork K. R. Sinsabaugh R. L. Zak D. R. (2002). The effects of long term nitrogen deposition on extracellular enzyme activity in an Acer saccharum forest soil. Soil Biol. Biochem. 34, 1309–1315. 10.1016/S0038-0717(02)00074-3

[B40] Schmidt M. W. Torn M. S. Abiven S. Dittmar T. Guggenberger G. Janssens I. A.. (2011). Persistence of soil organic matter as an ecosystem property. Nature 478, 49–56. 10.1038/nature10386 21979045

[B41] Shi G. Hou R. Li T. Fu Q. Wang J. Zhou W.. (2023). Effects of biochar and freeze–thaw cycles on the bacterial community and multifunctionality in a cold black soil area. J. Environ. Manag. 342:118302. 10.1016/j.jenvman.2023.118302 37267765

[B42] Sinsabaugh R. L. Hill B. H. Shah J. J. F. (2009). Ecoenzymatic stoichiometry of microbial organic nutrient acquisition in soil and sediment. Nature 462, 795–798. 10.1038/nature08632 20010687

[B43] Sparks D. L. Page A. L. Helmke P. A. Loeppert R. H. (2020). Methods of Soil Analysis, Part 3: Chemical Methods, Vol. 14. Hoboken, NJ: John Wiley and Sons.

[B44] Sradnick A. Ingold M. Marold J. Murugan R. Buerkert A. Joergensen R. G. (2014). Impact of activated charcoal and tannin amendments on microbial biomass and residues in an irrigated sandy soil under arid subtropical conditions. Biol. Fert. Soils 50, 95–103. 10.1007/s00374-013-0837-z

[B45] Tian J. Dungait J. A. J. Hou R. Deng Y. Hartley I. P. Yang Y.. (2024). Microbially mediated mechanisms underlie soil carbon accrual by conservation agriculture under decade-long warming. Nat. Commun. 15:377. 10.1038/s41467-023-44647-4 38191568 PMC10774409

[B46] Vu E. Schaumann G. Buchmann C. (2022). The contribution of microbial activity to soil-water interactions and soil microstructural stability of a silty loam soil under moisture dynamics. Geoderma 417:115822. 10.1016/j.geoderma.2022.115822

[B47] Wang B. An S. Liang C. Liu Y. Kuzyakov Y. (2021). Microbial necromass as the source of soil organic carbon in global ecosystems. Soil Biol. Biochem. 162:108422. 10.1016/j.soilbio.2021.108422

[B48] Wang J. Feng L. Palmer P. I. Liu Y. Fang S. Bosch H.. (2020). Large Chinese land carbon sink estimated from atmospheric carbon dioxide data. Nature 586, 720–723. 10.1038/s41586-020-2849-9 33116288

[B49] Wu H. Cai A. Dong W. Xing T. Xu M. Lu C. (2023). Nutrient stoichiometric management promotes carbon sequestration by improving microbial nutrient availability and metabolic efficiency in straw-amended soil. J. Soil. Sediment 23, 1182–1192. 10.1007/s11368-022-03396-5

[B50] Wu H. Cai A. Xing T. Huai S. Zhu P. Han X.. (2022). Integrated management of crop residue and nutrient enhances new carbon formation by regulating microbial taxa and enzymes. J. Integr. Agric. 21, 1772–1785. 10.1016/S2095-3119(21)63752-8

[B51] Zhang H. S. Wu X. H. Li G. Qin P. (2011). Interactions between arbuscular mycorrhizal fungi and phosphate-solubilizing fungus (*Mortierella* sp.) and their effects on *Kostelelzkya virginica* growth and enzyme activities of rhizosphere and bulk soils at different salinities. Biol. Fert. Soils 47, 543–554. 10.1007/s00374-011-0563-3

[B52] Zhang X. Dai G. Ma T. Liu N. Hu H. Ma W.. (2020). Links between microbial biomass and necromass components in the top- and subsoils of temperate grasslands along an aridity gradient. Geoderma 379:114623. 10.1016/j.geoderma.2020.114623

[B53] Zhang Y. Chen M. Zhao Y. Zhang A. Peng D. Lu F.. (2021). Destruction of the soil microbial ecological environment caused by the over-utilization of the rice-crayfish co-cropping pattern. Sci. Total Environ. 788:147794. 10.1016/j.scitotenv.2021.147794 34029817

[B54] Zhao Y. C. Wang M. Y. Hu S. J. Zhang X. D. Ouyang Z. Zhang Z. L.. (2018). Economics- and policy-driven organic carbon input enhancement dominates soil organic carbon accumulation in Chinese croplands. Proc. Natl. Acad. Sci. U. S. A. 115, 4045–4050. 10.1073/pnas.1700292114 29666318 PMC5910801

[B55] Zhou F. Zhang X. Ma S. Li Y. Zhu M. Zhang W.. (2023). Soil microbial necromass regulation of long-term fertilizer N retention influenced by maize stover mulching. Geoderma 433:116453. 10.1016/j.geoderma.2023.116453

[B56] Zhu X. Jackson R. D. Delucia E. H. Tiedje J. M. Liang C. (2020). The soil microbial carbon pump: from conceptual insights to empirical assessments. Glob. Change Biol. 26, 6032–6039. 10.1111/gcb.15319 32844509

[B57] Zhu Z. Fang Y. Liang Y. Li Y. Liu S. Li Y.. (2022). Stoichiometric regulation of priming effects and soil carbon balance by microbial life strategies. Soil Biol. Biochem. 169:108669. 10.1016/j.soilbio.2022.108669

