# Supplementary material for: Substrate stoichiometry and microbial metabolic preference drive the divergent accumulation of plant and microbial necromass carbon in cropland soils: evidence from a short-term experiment
Source: Front Microbiol. 2025 Jul 29;16:1619932. doi: 10.3389/fmicb.2025.1619932 (PMC12339551; doi:10.3389/fmicb.2025.1619932)
Supplement: Supplementary file 1 [file Data_Sheet_1.docx]

**Supplementary material for:**

**Substrate stoichiometry and microbial metabolic preference drive the divergent accumulation of plant and microbial necromass carbon in cropland soils**

Hongliang Wu^1^, Luming Wang^1^, Xiuping Liu^1^, Wenyan Wang^1^, Changai Lu^2^ and Wenxu Dong^1^*

^1^Hebei Key Laboratory of Soil Ecology, Center for Agricultural Resources Research, Institute of Genetics and Developmental Biology, Chinese Academy of Sciences, Shijiazhuang 050022, China

^2^Institute of Agricultural Resources and Regional Planning, Chinese Academy of Agricultural Sciences, Beijing 100081, China

* Correspondence: Wenxu Dong E-mail address: [dongwx@sjziam.ac.cn](mailto:dongwx@sjziam.ac.cn)

Table S1 Introduction of carbon-, nitrogen-, and phosphorus-acquiring enzymes studied.

| Group | Function | Enzyme | Substrate | Abbr |
| --- | --- | --- | --- | --- |
| C acquisition | Hydrolysis of Sugar | β-glucosidase | 4-Methylumbelliferyl β-Dglucopyranoside 6.77 mg/100 ml | BG |
|  | Hydrolysis of Cellobiose | cellobiohydrolase | 4-methylumbelliferyl-β-d-cellobioside 10.01 mg /100 ml | CBH |
| N acquisition | Hydrolysis of protein | L-leucine aminopeptidase | L-Leucine-7-amido-4-methylcoumarin hydrochloride 6.5 mg/100 ml | LAP |
|  | Hydrolysis of chitin | β-N-acetylglucosaminidase | 4-Methylumbelliferyl N-acetyl-β-Dglucosaminide 7.59 mg/100 ml | NAG |
| P acquisition | Hydrolysis of organic P | acid phosphatase | 4-Methylumbelliferyl phosphate 5.12 mg/100 ml | AP |
| MUB standard |  |  | 4-Methylumbelliferone 1.76 mg/100 ml Dissolve in 1 ml acetone first | MUB |
| AMC standard |  |  | 7-Amino-4-methylcoumarin 1.75 mg/100 ml Dissolve in 1 ml acetone first | AMC |

Table S2 Basic chemical properties in the top- and subsoil at different sampling periods.

| Soil indicators | Sampling time | Soil layer | CK | NPS0 | NPS1 | NPS2 | NPS3 |
| --- | --- | --- | --- | --- | --- | --- | --- |
| SOC (g kg^-1^) | Day 30 | topsoil | 11.39±0.29b* | 12.51±0.20a* | 12.60±0.12a* | 12.35±0.25a* | 12.26±0.17a* |
|  |  | subsoil | 9.59±0.01c | 10.66±0.18b | 10.88±0.11ab | 11.34±0.31a | 10.87±0.10ab |
|  | Day 90 | topsoil | 11.21±0.16b* | 12.37±0.29a* | 12.83±0.12a* | 12.91±0.28a* | 12.77±0.11a* |
|  |  | subsoil | 9.26±0.16b | 11.02±0.30a | 12.17±0.86a | 11.58±0.46a | 11.35±0.34a |
|  | Day 150 | topsoil | 11.20±0.29b* | 12.80±0.23a* | 13.28±0.16a* | 12.51±0.21a* | 12.54±0.13a* |
|  |  | subsoil | 9.40±0.31b | 10.87±0.48a | 11.58±0.22a | 11.12±0.29a | 11.59±0.07a |
| NO_3_^-^-N (mg kg^-1^) | Day 30 | topsoil | 28.62±2.01c* | 29.83±5.59c* | 32.26±7.74c* | 37.81±2.76b* | 51.62±2.47a* |
|  |  | subsoil | 15.32±2.89c | 16.09±1.83c | 15.83±1.58c | 26.32±3.90b | 34.87±6.90a |
|  | Day 90 | topsoil | 19.63±0.19c* | 25.33±0.03b* | 27.19±0.40b* | 34.19±0.15b* | 43.45±0.64a* |
|  |  | subsoil | 12.19±0.09b | 9.15±0.32b | 19.14±0.36a | 22.19±0.17a | 21.23±0.24a |
|  | Day 150 | topsoil | 28.96±0.16b* | 32.26±0.35b* | 35.45±0.34a* | 35.79±0.19a* | 36.21±0.53a* |
|  |  | subsoil | 15.04±0.18b | 12.23±0.67b | 10.88±0.40b | 19.45±0.29a | 22.15±0.19a |
| NH_4_^+^-N (mg kg^-1^) | Day 30 | topsoil | 3.07±0.25c* | 7.66±0.63b* | 7.20±0.46b | 8.89±0.51b | 10.35±0.83a |
|  |  | subsoil | 1.64±0.10c | 5.74±0.28b | 6.90±1.18b | 7.66±0.43b | 11.21±0.61a |
|  | Day 90 | topsoil | 3.52±0.29b* | 6.17±0.40a* | 8.30±0.55a | 8.09±0.57a | 8.18±0.67a |
|  |  | subsoil | 2.38±0.11b | 4.37±0.23b | 7.56±1.11a | 7.82±0.21a | 8.76±0.80a |
|  | Day 150 | topsoil | 2.80±0.34b* | 6.89±0.21a* | 6.85±0.33a* | 5.96±0.14a | 6.42±0.39a |
|  |  | subsoil | 1.03±0.13c | 3.92±0.25b | 5.32±0.42a | 5.70±0.20a | 6.19±0.33a |
| SAP (mg kg^-1^) | Day 30 | topsoil | 18.81±0.22d* | 18.21±0.34d* | 22.23±0.28c* | 27.92±0.39b* | 32.68±1.25a* |
|  |  | subsoil | 2.87±0.15e | 4.58±0.19d | 6.47±0.11c | 10.03±0.22b | 14.42±1.22a |
|  | Day 90 | topsoil | 19.60±0.20c* | 19.18±0.44c* | 20.75±0.36c* | 25.84±0.56b* | 27.04±0.58a* |
|  |  | subsoil | 3.47±0.17c | 3.56±0.06c | 5.78±0.44b | 7.03±0.29a | 6.84±0.15a |
|  | Day 150 | topsoil | 22.23±0.20b* | 19.18±0.36b* | 21.68±3.05b* | 23.650.22ab* | 26.81±1.06a* |
|  |  | subsoil | 2.96±0.05b | 3.74±0.25b | 4.53±0.44b | 6.38±0.38a | 7.72±1.24a |

Different lowercase letters in horizontal rows mean significant differences among NPS0–NPS3 in the top- and subsoil, respectively (*P* < 0.05). An asterisk indicates significant difference between the top- and subsoil (*p* < 0.05). The number after “±” is the standard error of the mean (*n* = 3). SOC, soil organic carbon; NO_3_^-^-N/ NH_4_^+^-N, nitrate nitrogen/ammonium nitrogen; SAP, soil available phosphorus; CK, soil only; NPS0, soil + straw; NPS1–NPS3, soil + straw + incremental nitrogen, phosphorus, and sulfur addition.

Table S3 Stoichiometric ratios of the activities for carbon, nitrogen and phosphorus acquisition enzymes.

| Depth | Treatment | BG+CBH/LAP+NAG | | |  | BG+CBH/AP | | |  | LAP+NAG/AP | | |
| --- | --- | --- | --- | --- | --- | --- | --- | --- | --- | --- | --- | --- |
|  |  | Day 30 | Day 90 | Day 150 |  | Day 30 | Day 90 | Day 150 |  | Day 30 | Day 90 | Day 150 |
| 0-20  cm | CK | 2.02±0.01a^A^ | 1.94±0.02a^A^ | 2.03±0.04a^A^ |  | 3.14±0.11b^A^ | 2.77±0.01d^B^ | 3.11±0.14a^A^ |  | 1.56±0.11b^A^ | 1.48±0.01b^A^ | 1.53±0.14b^A^ |
|  | NPS0 | 2.05±0.05a^A^ | 1.95±0.03a^A^ | 2.02±0.05a^A^ |  | 3.34±0.05a^A^ | 3.38±0.06a^A^ | 3.17±0.07a^A^ |  | 1.63±0.05a^A^ | 1.73±0.06a^A^ | 1.57±0.07ab^B^ |
|  | NPS1 | 1.91±0.06b^A^ | 1.81±0.01b^B^ | 1.97±0.01a^A^ |  | 3.30±0.09ab^A^ | 3.29±0.10ab^A^ | 3.16±0.05a^A^ |  | 1.73±0.09a^A^ | 1.72±0.10a^A^ | 1.61±0.05a^B^ |
|  | NPS2 | 1.75±0.03c^B^ | 1.72±0.06c^B^ | 1.96±0.07a^A^ |  | 2.82±0.05c^B^ | 3.17±0.08b^A^ | 3.14±0.01a^A^ |  | 1.67±0.05a^A^ | 1.75±0.08a^A^ | 1.60±0.01a^B^ |
|  | NPS3 | 1.78±0.01c^A^ | 1.73±0.01c^A^ | 1.82±0.02b^A^ |  | 2.94±0.03c^A^ | 2.91±0.07c^A^ | 2.88±0.08b^A^ |  | 1.65±0.03a^A^ | 1.69±0.07a^A^ | 1.58±0.08a^B^ |
| 20-40cm | CK | 2.00±0.02a^A^ | 1.79±0.03b^B^ | 1.79±0.02ab^B^ |  | 2.94±0.02ab^A^ | 2.77±0.04b^B^ | 2.83±0.02a^B^ |  | 1.47±0.02a^B^ | 1.38±0.04b^B^ | 1.58±0.02a^A^ |
|  | NPS0 | 1.91±0.07a^A^ | 1.91±0.02a^A^ | 1.84±0.04a^A^ |  | 2.70±0.06b^B^ | 2.76±0.04b^B^ | 2.94±0.03a^A^ |  | 1.41±0.06a^B^ | 1.44±0.04b^B^ | 1.60±0.03a^A^ |
|  | NPS1 | 1.96±0.02a^A^ | 1.89±0.05ab^A^ | 1.87±0.05a^A^ |  | 2.70±0.14b^B^ | 3.18±0.08a^A^ | 2.99±0.06a^A^ |  | 1.38±0.14a^B^ | 1.68±0.08a^A^ | 1.60±0.06a^A^ |
|  | NPS2 | 1.92±0.02a^A^ | 1.85±0.03ab^A^ | 1.75±0.01b^B^ |  | 2.69±0.05b^B^ | 3.02±0.01a^A^ | 2.86±0.02a^A^ |  | 1.40±0.05a^B^ | 1.63±0.01a^A^ | 1.63±0.02a^A^ |
|  | NPS3 | 2.03±0.06a^A^ | 1.81±0.06ab^B^ | 1.73±0.02b^B^ |  | 3.05±0.14a^A^ | 2.97±0.07a^A^ | 2.74±0.01b^B^ |  | 1.50±0.14a^A^ | 1.64±0.07a^A^ | 1.58±0.01a^A^ |

Different lowercase letters in vertical column mean significant differences among NPS0–NPS3, different uppercase letters in horizontal rows mean significant differences among different sampling times (*p* < 0.05). The number after “±” is the standard error of the mean (*n* = 3). BG, β-glucosidase; CBH, cellobiohydrolase; LAP, L-leucine aminopeptidase; NAG, β-N-acetylglucosaminidase; AP, acid phosphatase. CK, soil only; NPS0, soil + straw; NPS1–NPS3, soil + straw + incremental nitrogen, phosphorus, and sulfur addition.





Figure S1 Evolution of lignin phenols (vanillyl, syringyl and cinnamyl) over time under different substrate stoichiometric ratios in the top- and subsoil. Different lowercase or uppercase letters above the bar mean significant differences among NPS0–NPS3 in the top- and subsoil, respectively (*p* < 0.05). CK, soil only; NPS0, soil + straw; NPS1–NPS3, soil + straw + incremental nitrogen, phosphorus, and sulfur addition





Figure S2 The soil moisture content on days 30, 90 and 150 under different substrate stoichiometric ratios in the top- and subsoil during the maize season. Different lowercase or uppercase letters mean significant differences among NPS0–NPS3 in the top- and subsoil, respectively (*p* < 0.05). An asterisk indicates significant difference between the top- and subsoil (*p* < 0.05). CK, soil only; NPS0, soil + straw; NPS1–NPS3, soil + straw + incremental nitrogen, phosphorus, and sulfur addition.





Figure S3 Activities of cellobiohydrolase (CBH) and β-N-acetylglucosaminidase (NAG) under different nutrient addition levels at three sampling times in the top- and subsoil. An asterisk indicates significant difference between the top- and subsoil (*p* < 0.05). CK, soil only; NPS0, soil + straw; NPS1–NPS3, soil + straw + incremental nitrogen, phosphorus, and sulfur addition.





Figure S4 Alpha diversity of bacteria and fungus under different nutrient addition levels at three sampling times in the top- and subsoil during the maize season. Different lowercase or uppercase letters mean significant differences among NPS0–NPS3 in the top- and subsoil, respectively (*p* < 0.05). An asterisk indicates significant difference between the top- and subsoil (*p* < 0.05). CK, soil only; NPS0, soil + straw; NPS1–NPS3, soil + straw + incremental nitrogen, phosphorus, and sulfur addition.





**Figure S5** Principal coordinate analysis (PCoA) based on OTU level for bacterial (a, b) and fungal (c, d) communities. The distance between samples (based on Bray-Curtis dissimilarity) reflects the dispersion degree of microbial communities. Percentages indicate the explanatory power of each principal coordinate axis for compositional variation. Closer sample points indicate more similar community compositions. The circle represents the topsoil sample, and the square represents the subsoil sample. Statistical differences reflect variations between topsoil and subsoil. CK, soil only; NPS0, soil + straw; NPS1–NPS3, soil + straw + incremental nitrogen, phosphorus, and sulfur addition; T, topsoil; S, subsoil.


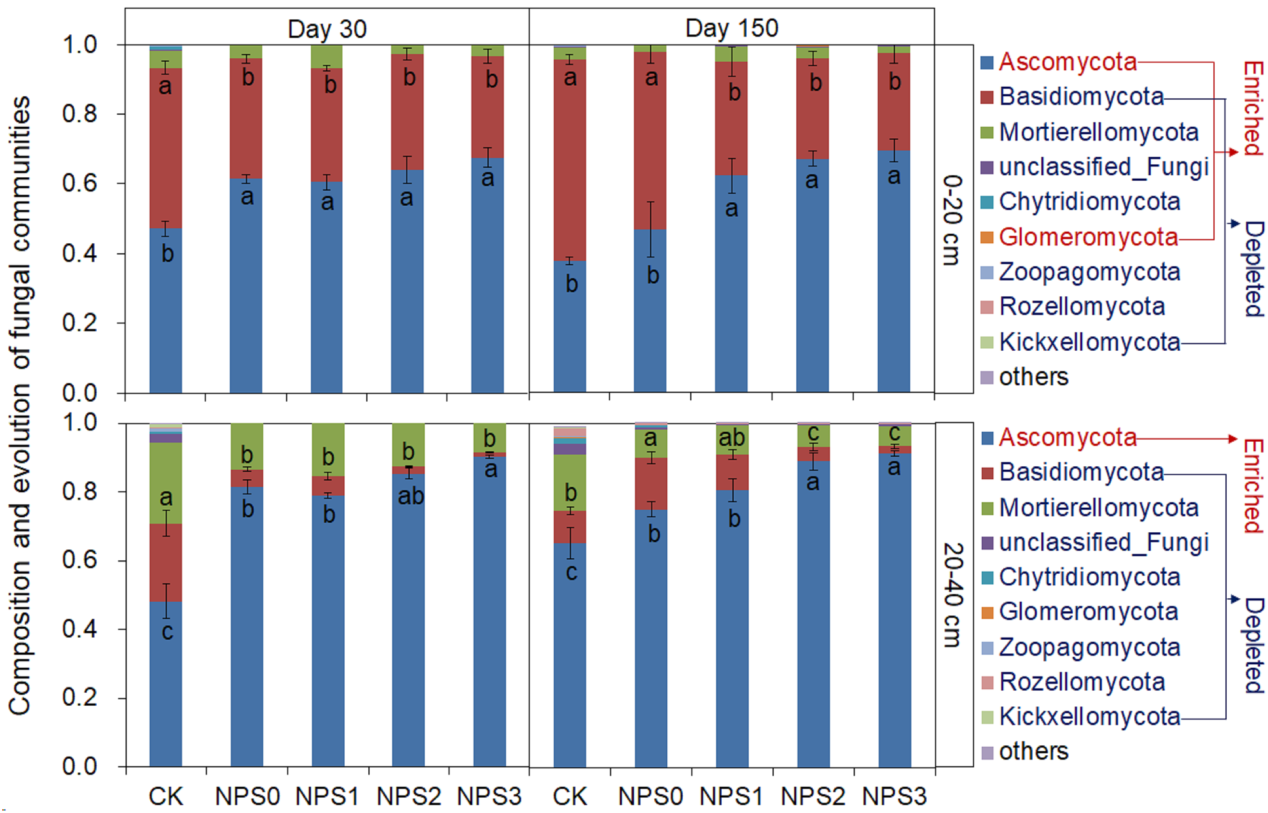


Figure S6 Composition of fungal communities (on phylum level) on days 30 and 150 under different substrate stoichiometric ratios in the top- and subsoil. Different lowercase letters above the error bar mean significant differences among NPS0–NPS3 (*p* < 0.05). CK, soil only; NPS0, soil + straw; NPS1–NPS3, soil + straw + incremental nitrogen, phosphorus, and sulfur addition.
